# The Effect of Acidic Immersion Media on the Flexural Properties of a High-Performance Fiber-Reinforced CAD/CAM Technopolymer

**DOI:** 10.3390/polym17091216

**Published:** 2025-04-29

**Authors:** Hanin E. Yeslam, Hazzaa H. Alqahtani, Aws M. Filemban, Sultan O. Jiffri, Abeer K. Tashkandi

**Affiliations:** 1 Department of Restorative Dentistry, Faculty of Dentistry, King Abdulaziz University, Jeddah P.O. Box 80200, Saudi Arabia; btashkndee@kau.edu.sa; 2 Advanced Technology Dental Research Laboratory, King Abdulaziz University, Jeddah P.O. Box 80209, Saudi Arabia; 3 Faculty of Dentistry, King Abdulaziz University, Jeddah P.O. Box 80200, Saudi Arabia; halgahtani0025@stu.kau.edu.sa (H.H.A.); afilemban0004@stu.kau.edu.sa (A.M.F.); sjiffri0002@stu.kau.edu.sa (S.O.J.)

**Keywords:** fiber-reinforced composites, CAD/CAM, flexural strength, acidic immersion, GERD, mechanical properties, restorative dentistry, dental restorations

## Abstract

Introduction: High-performance fiber-reinforced technopolymers for computer-aided design/computer-aided manufacturing (CAD/CAM) of dental restorations offer superior durability and strength. However, exposure to acidic solutions may adversely affect these mechanical properties. Objective: This study aimed to evaluate the flexural properties of a high-strength commercially available CAD/CAM fiber-reinforced dental material in response to water, cola, and artificial gastric acid solutions. Method: Forty bar-shaped specimens (1 × 4 × 13 mm) were fabricated from a pre-polymerized glass fiber-reinforced composite (Trilor disks, Bioloren, Saronno, Italy). Ten specimens were randomly selected for baseline testing. The remaining specimens were subdivided into three groups based on the storage media (n = 10): artificial gastric acid solution, Coca-Cola, and deionized water (37 °C, 48 h). Mean flexural strengths and moduli were statistically compared at a significance level of *p* < 0.05. Results: No statistically significant change in flexural strength was observed after immersion in the different media. However, there was a statistically significant decrease in the flexural modulus after storage for 48 h, regardless of pH. Conclusion: Fiber-reinforced CAD/CAM technopolymers show promising strength stability in response to varying pH conditions. However, further studies are needed to investigate the material’s long-term strength stability.

## 1. Introduction

Computer-aided design/computer-aided manufacturing (CAD/CAM) technology allows for the custom creation of esthetically pleasing dental restorations with good precision and mechanical properties [1,2]. A wide range of esthetic materials, including fiber-reinforced composites for dental CAD/CAM, offer advantages like crack resistance, ease of use, and good performance [3]. Fiber-reinforced composite (FRC) dental materials consist of a matrix that absorbs external stresses and transfers them to dispersed reinforcement fibers, typically glass fibers, and an interphase region in the set material [4,5,6]. The fibers are included in the polymeric composite structure for their high stiffness-to-weight ratio, specific strength, and chemical stability in environments with a pH range between 4 and 11 [6,7]. These fibers are thought to enhance the final restoration’s strength [8]. Light-weight novel polymeric FRC CAD/CAM materials can be used for the fabrication of durable frameworks for removable and fixed partial dentures (RPDs and FPDs, respectively), periodontal stents, implant-supported restorations, and dental pontics [8,9]. FRC CAD/CAM materials are typically recommended only for dental restoration framework fabrication, and are not suitable for milling restorations to full contour, due to their fibers’ potential to irritate adjacent gingival soft tissue [9,10]. Recently, a novel FRC technopolymer (Trilor (Bioloren, Saronno, Italy)) has been introduced for the CAD/CAM fabrication of both monolithic final restorations, RPD clasps and frameworks, and metal-free frameworks of FPDs [2,11].

Various intraoral factors, including changes in acidity and temperature, can affect the strength, lifespan, and durability of biomaterials used for CAD/CAM fabrication of fixed partial dentures (FPDs) [12]. Long-term exposure to intraoral acidic environments can eventually lead to dental erosion and adversely affect dental restorations [13]. External acidic sources include beverages like citrus juices, flavored drinks, caffeinated beverages, and wines. Internal sources involve stomach acid exposure from vomiting or disorders like gastroesophageal reflux disease (GERD), bulimia nervosa, and gastric dysfunction [14]. Assessing the effects of intraoral acids on the physical and mechanical properties of high-strength CAD/CAM materials is vital for assessing their long-term performance and clinical success [15]. The stability of two relatively new FRC materials for CAD/CAM fabricated restorations (the technopolymer Trilor and the FRC Trinia (BICON, Boston, MA, USA)) was assessed using immersion assays, which indicated their plausible stability when exposed to artificial saliva at various pH levels [4].

One of the most important mechanical factors affecting the durability of dental restorations is flexural strength, which refers to the maximum bending stress that a dental material can withstand before it deforms or fractures. Adequate flexural strength is essential for creating long-lasting restorations that can resist occlusal forces and functional loads [4,16]. The elastic modulus of dental materials, on the other hand, helps to determine their intraoral flexural behavior, as a higher modulus signifies a stiffer material that resists deformation and provides support to the restored tooth [17]. The higher flexural strength of FRCs for dental CAD/CAM restorations has also been demonstrated in previous studies in the literature when compared to fiber-reinforced and non-reinforced dental PEEK materials [18,19]. A finite element analysis (FEA) showed that FRCs and PEEK CAD/CAM materials possess a lower modulus of elasticity than lithium disilicate and zirconia, allowing them to be used create biomimetic restorations able to absorb forces in a similar way to natural teeth [20].

Limited conflicting evidence in the literature exists regarding the effect of acidic media on the flexural properties of CAD/CAM dental materials. Some studies have investigated the impact of short-term exposure to intrinsic and extrinsic acidic media on composite CAD/CAM materials, particularly gastric acid and Coca-Cola, and concluded no significant effects on the flexural strength of these materials [14,21,22,23,24]. This was also true for a novel experimental FRC material that underwent various acid and thermal short-term aging protocols [25]. On the other hand, some studies have demonstrated a decrease in the flexural strength and microhardness of composite CAD/CAM blocks after prolonged storage in different acids [24,26]. The flexural strength of an FRC CAD/CAM material (Trinia) significantly decreased after being exposed to water mixed with methyl ethyl alcohol and to water mixed with ethanol for 1 day, and then was further reduced after 7 days of exposure [18]. In a previous study, the detrimental effect of thermal aging on the flexural strength of an FRC technopolymer (Trilor) was found to be significant. However, the pH of the immersion solution was not included in the investigation [2].

To the best of the authors’ knowledge, no studies have evaluated the flexural strength of technopolymer-based FRC CAD/CAM material exposed to gastric acid and Coca-Cola for durations longer than 24 h. Thus, it is necessary to reassess the impacts of gastric acid and Coca-Cola on the flexural properties of FRC CAD/CAM technopolymer blocks over a period longer than 24 h. This study aimed to investigate the flexural properties of a novel commercially available high-strength FRC technopolymer for CAD/CAM fabrication of full-contour definitive restorations in response to an intrinsic acidic medium (artificial gastric acid) and a commonly encountered extrinsic (cola-based beverage) acidic medium. The null hypothesis for this study was that there would be no significant change in the flexural strength and elastic modulus of FRC technopolymer CAD/CAM blocks following storage in water, artificial gastric acid, and Coca-Cola for 48 h.

## 2. Materials and Methods

This in vitro study tested the flexural strength and modulus of a commercially available, pre-polymerized glass fiber-reinforced technopolymer composite (TD) for CAD/CAM fabrication of definitive restorations. The tested material can be used for the milling of monolithic restorations, requires only polishing, and does not need firing after milling. Table 1 details the composition and mechanical properties of the tested material, as supplied by the manufacturer.

A total of 40 bar-shaped specimens measuring 13 ± 1 × 4 × 1 mm were fabricated from the TD material. After finishing and polishing, 10 specimens were randomly selected for initial flexural testing (3-point bending test in universal testing machine, baseline readings (TDB)). Depending on the storage media, the remaining 30 specimens were randomly divided into 3 subgroups (n = 10), as follows: lab-prepared artificial gastric acid (TDA), acid-containing soda drink (Coca-Cola, Aujan Coca-Cola Beverages Company, KSA, Dammam, Saudi Arabia) (TDC), and deionized water (TDW) in an incubator (37 °C, 48 h). After 24 h, the storage medium was refreshed, and the specimens were then returned to the incubator for another 24 h, to reach a total storage time of 48 h. Afterwards, all specimens underwent a 3-point bending test in a universal testing machine. The mean flexural strengths and moduli of the groups were statistically compared.

### 2.1. Specimen Preparation

Forty bar-shaped specimens (13 ± 1 × 4 × 1 mm) were prepared by sectioning the TD disk into plates, and then into bars, to reach the required dimensions. This was completed using a precision low-speed saw cutting machine (TechCut 4™ precision low-speed saw, Allied, Edmond, OK, USA) with a diamond-coated saw blade (lapidary saw blade with super-thin rim, outer diameter—100 mm, arbor hole—12.7 mm, core thickness—0.23 mm, Jingling, Yangzhou, China), under constant water cooling. Rough finishing of extremely uneven areas was performed using 220-grit, and then 400-grit, silicon carbide (SiC) paper. Fine finishing and polishing of the specimen top and bottom surfaces were performed using SiC paper with grits of 600, 800, and 1200 (Middle East factory, MEA, Jeddah, Saudi Arabia) on a rotary polishing machine (Metaserv; Buehler, Düsseldorf, Germany), under copious water irrigation. The final dimensions were confirmed using a digital caliper (vernier caliper 200 mm/8 in, Hi-Wendy, New Taipei, Taiwan). All specimens were rinsed with deionized water for 10 s after polishing to remove debris.

### 2.2. Specimens Immersion

The baseline group specimens (TDB) (n = 10) were rinsed with deionized water, then pat-dried, and immediately tested in the universal testing machine. Each specimen in the TDW group was immersed in 5 mL of deionized water. The TDA group specimens were immersed in lab-prepared artificial gastric acid (each specimen in 5 mL hydrochloric acid (HCL) solution, 0.113 wt% HCl). Each TDC group specimen was immersed in 5 mL of a commercially available cola drink containing phosphoric acid. The immersed specimens were stored in an incubator for 48 h at 37 °C (water and immersion solutions were replaced at the 24 h mark). This immersion protocol corresponds to around 5 years of intraoral exposure to acidic media (30 min/exposure 3 times/day) [14]. The immersion solutions, their pH, and their respective constituents are detailed in Table 2.

### 2.3. Flexural Strength and Modulus Testing

**Figure 1:**
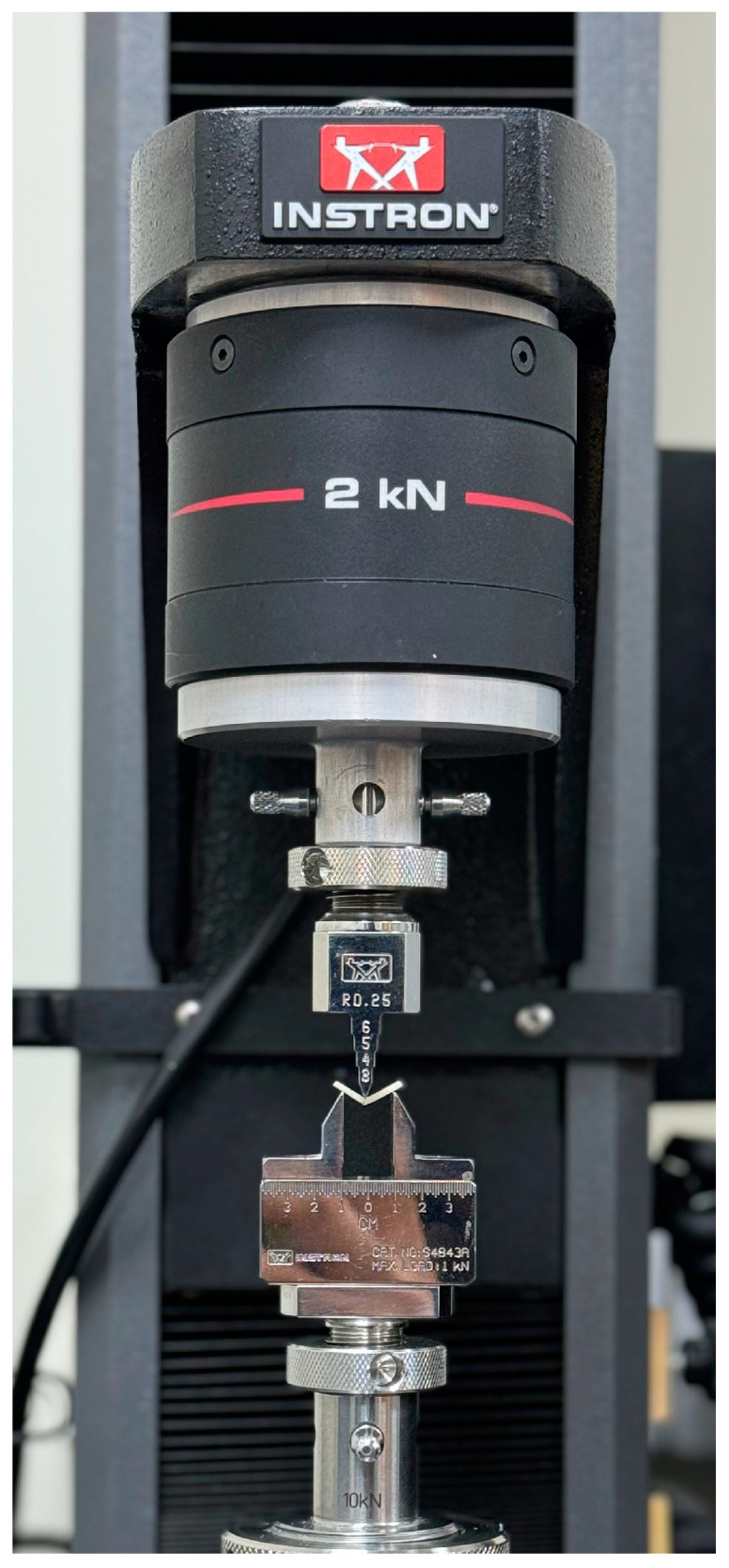
A TD specimen loaded to fracture in the universal testing machine.

All test specimens were subjected to a 3-point bending test using a universal testing machine (5940 Series, Instron, Norwood, MA, USA), with a crosshead speed of 1 mm/min and a 2 kN load cell. The span was set at 10 mm. All specimens were loaded until complete deformation. A representative example of a fractured specimen undergoing the 3-point bending test in the universal testing machine is demonstrated in Figure 1.

The following equations were used for the calculation of flexural strength ($F_{s}$) (MPa) and modulus ($E_{flex})$(GPa):

$$F_{s}=\frac{3fl}{2wh^{2}}$$

$$E_{flex}=\frac{l^{3}f}{4wh^{3}d}$$

where *f* is the maximum applied load, in newtons (N); *l* is the distance, in millimeters (mm), between the supports; *w* is the width of the specimen, in millimeters (mm); and *h* is the height of the specimen, in millimeters (mm).

### 2.4. Statistical Analysis

Calculations of sample size and power were completed using the G*Power software, Version 3.1.9.7 (G*Power by Franz Faul, Universität Kiel, Kiel, Germany). A total sample size of N = 40 was adopted in the study to detect large differences between samples (f = 0.55) and achieve a 0.8 study power at an alpha-error probability of 0.05.

The results were statistically analyzed using the statistical software SPSS (SPSS, Version 22, IBM Corporation, New York, NY, USA). In each group, the following descriptive statistical parameters of flexural strength and modulus were calculated: mean, standard deviation, minimum, and maximum. The Kolmogorov–Smirnov test was used to confirm the normal distribution of the results at a significance level of 0.05. One-way analysis of variance (ANOVA) was used to compare the mean $F_{s}$ and $E_{flex}$ values between the different groups. Post hoc Tukey’s HSD pairwise comparison was utilized to identify significant differences between pairs of immersed groups. A 5-percent significance level was used for all comparisons.

## 3. Results

**Figure 2:**
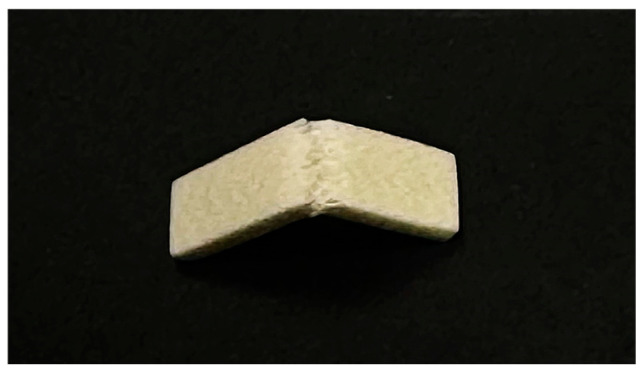
Fractured specimen after 3-point bending test.

All specimens were visually inspected following the flexural test. Upon inspection, all tested specimens showed glass fibers protruding from the two parts of the specimen at the fracture line after flexural loading until fracture. Figure 2 shows a representative fractured sample with the glass fibers protruding at the fracture line.

### 3.1. Flexural Strength Results

**Figure 3:**
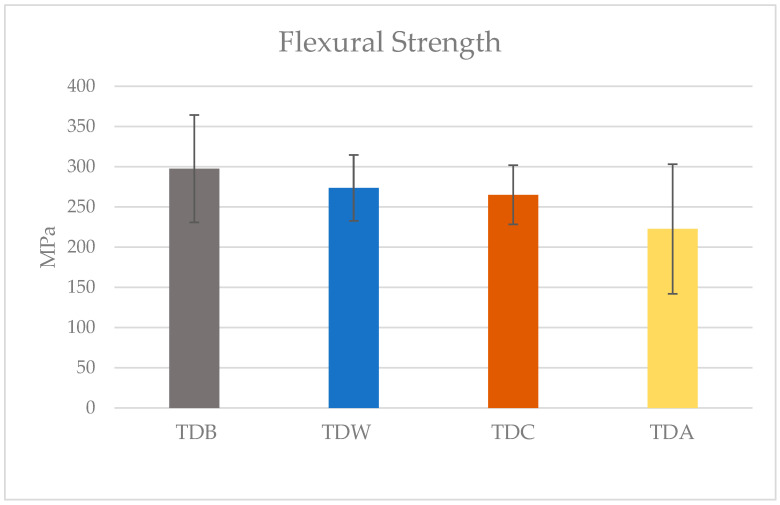
Flexural strength means and standard deviations of the groups at baseline and after immersion in the different media. TDB is the baseline group, TDW is the water-immersion group, TDC is the cola-immersion group, and TDA is the acid-immersion group.

The highest mean flexural strength was found in the TDB group (297.54 ± 66.68 MPa), followed by the TDW (273.49 ± 41.02 MPa), TDC (265 ± 36.81 MPa), and TDA (241.38 ± 27.15 MPa) groups, respectively. The Kolmogorov–Smirnov test of normality showed that the data did not differ significantly from that of a normal distribution (*p* = 0.79). Therefore, parametric testing was used to compare the groups. One-way ANOVA showed that there were no statistically significant differences in the mean flexural strength between the different immersion groups (*p* = 0.66). The mean flexural strengths and standard deviations are demonstrated in Figure 3.

### 3.2. Flexural Modulus Results

After confirming the normal distribution of the results using the Kolmogorov–Smirnov test (*p* = 0.56), a one-way ANOVA test comparing the mean flexural moduli of the groups revealed the presence of significant differences between them (*p* < 0.001). A post hoc Tukey HSD pairwise comparison revealed a significantly higher modulus in the TDB group (13.15 ± 3.53 GPa), than in the TDW (10.53 ± 1.46 GPa), TDC (10.54 ± 1.59 GPa), and TDA (8.84 ± 1.16 GPa) groups (*p* = 0.047, *p* = 0.47, and *p* < 0.001, respectively). However, there were no statistically significant differences between the immersion groups (*p* = 0.3).

**Figure 4:**
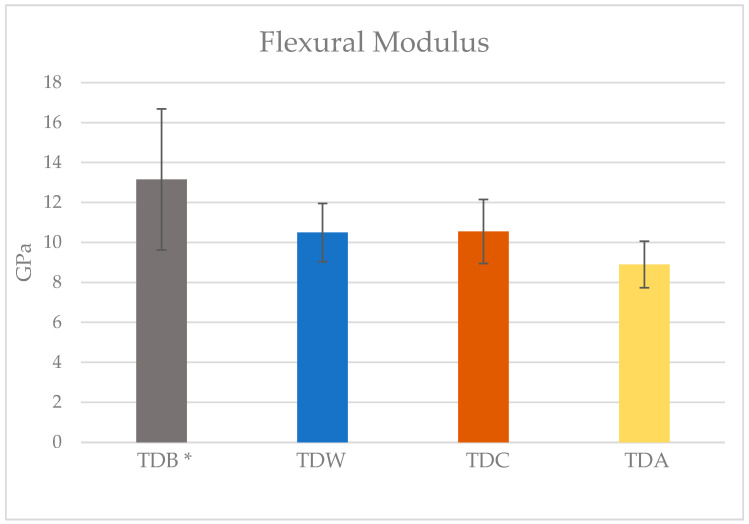
Flexural modulus means and standard deviations of the TD material at baseline and after immersion in the different media. TDB is the baseline group, TDW is the water-immersion group, TDC is the cola-immersion group, and TDA is the acid-immersion group. * Statistically significant difference at *p* < 0.05.

The mean flexural moduli for the TD material baseline and immersion groups are detailed in Figure 4.

## 4. Discussion

Flexural strength plays a crucial role in assessing the strength of definitive FPD and restoration materials. By evaluating flexural strength, the ability of these materials to withstand applied forces and resist deformation or fracture under bending stress can be quantified [27]. Computer-aided design/computer-aided manufacturing (CAD/CAM) systems enable efficient modeling and production, using composite hybrid materials with improved mechanical properties and repairability. A restorative material with a high strength and an elastic modulus that resembles tooth structure is vital to the fabrication of biomimetic restorations that can absorb forces in a similar way to natural teeth [20]. The current study aimed to evaluate the effect of water storage and acidic media on the flexural strength and modulus of a commercially available high-strength CAD/CAM dental fiber-reinforced technopolymer intended for use in the fabrication of monolithic full-contour definitive dental restorations (TDs: Trilor disks, Bioloren, Saronno, Italy). The material can be milled into full-contour restorations or into metal-free frameworks to be veneered by esthetic ceramic or composite, without the need for post-milling firing or sintering [11].

In this study, the specimens were immersed in a simulated gastric acid solution and a Coca-Cola beverage. These immersion media were selected based on the fact that they are commonly encountered, allowing for a meaningful comparison of the study results with previously published research results [4,14,28,29,30,31]. The current dietary habits of patients, including heavy consumption of acidic carbonated beverages, have a huge impact on the esthetic and physical durability of dental restorations [31]. Due to a significant rise in the consumption of Coca-Cola, an extremely popular beverage worldwide, across different age groups [31,32], it was chosen as a storage medium in the current study to examine how it could affect the performance of Trilor CAD/CAM technopolymer. Moreover, the prevalence of GERD and eating disorders that impact the pH of the oral environment has increased significantly over the years [4,33]; therefore, the current study aimed to investigate the impact of such challenges on the flexural behavior of the new Trilor technopolymer in final dental restorations. Based on the results of the current study, the null hypothesis—that there would be no significant change in the flexural strength and modulus of the TD material following storage in water, artificial gastric acid, and Coca-Cola for 48 h—was partially accepted, as there were no statistically significant differences in flexural strength due to immersion in the different media. On the other hand, there was a statistically significant decrease in the flexural modulus of TD specimens after immersion in the different media. However, there were no significant differences in that decrease between the different immersion media.

Maintaining a high flexural strength value is desirable, as it indicates a material’s ability to withstand forces in the oral environment, such as chewing and biting, without experiencing deformation [34]. A high flexural modulus indicates a stiffer material, while a low modulus reflects a material that is liable to deformation, which may result in faster failure of the final restoration [2,12]. In the current study, the three-point bending test was conducted using small-sized specimens similar to those used in a previous mini flexural strength study [2]. According to ISO 4049 standard, polymer-based restorative dental materials are required to have a minimum flexural strength of 80 MPa [35]. The flexural strength values attained in the current study were higher than the required minimum, but lower than those stated by the material manufacturer. Additionally, the values were similar to those reported by previous mini flexural test studies. The difference in flexural strength value between the manufacturer and the current study can also be attributed to the different testing methodologies (three-point vs. biaxial). Both testing modalities were deemed comparable in a previous study [36], so the three-point mini flexural bending test was adopted in the current study. The flexural modulus of a smaller specimen may appear higher than that of a larger one, due to the concentration of stress over a smaller area. It is essential to take this effect into account when analyzing test results, and to ensure that the testing conditions reflect real clinical situations [37]. Additionally, it has been reported that biaxial flexural testing is an accurate methodology for materials that exhibit minimal plastic deformation, such as ceramics [36]. The smaller specimen size used in this method may have led to higher values reported by manufacturers compared to those obtained in the current study, which utilized a three-point bending test [37]. In the three-point bending test, stresses are concentrated at a single point on the specimen, resulting in earlier failure. All specimens were tested after finishing and polishing. Finishing and polishing eliminate the surface compressive stress layer, which can create micro-cracks, leading to restoration failure. Therefore, it is essential to carefully control this process to maintain the esthetic restorative material’s strength [38]. The effect of different finishing and polishing methods with different micro-crack-forming potentials was controlled in the current study by allowing all specimens to undergo the exact same finishing procedures, to maintain standardization in specimen preparation.

In the current study, all specimens were immersed in acidic media for 48 h at 37 °C. The 48 h immersion period in acidic media for the specimens was chosen based on previous dental material research, to replicate the effects of acidic environments. A 24 h immersion has been found to produce some changes in other CAD/CAM hybrid materials, according to previous studies [14,39]. On the other hand, a 24 h immersion has not been found to produce significant changes in other composite CAD/CAM materials [23,24,40]. Previous research, such as that by Bechir et al. [4], has shown that a period of around 48 h is sufficient to observe significant changes in the physical and chemical properties of dental FRC materials when exposed to acidic solutions, and to demonstrate initial degradation processes. A 24 h erosive immersion study protocol was reported to correspond to two and a half years of intraoral exposure to acids, as teeth are exposed to acids three times/day for a duration of 30 min/exposure [14,41]. Therefore, the 48 h immersion protocol employed in the current study resembles the effects of five years of intraoral exposure. Therefore, a 48 h immersion period was chosen for the current study to evaluate the effect of this longer duration.

After 48 h of immersion in the different media, the statistical analysis of mean flexural strength revealed the absence of any statistically significant differences in the material. These results are in accordance with the findings from a study by Alnsour et al. [21], which investigated the effect of 24 h and 96 h storage of polymer-infiltrated ceramic network and composite CAD/CAM materials in artificial gastric acid and Coca-Cola on their flexural strength and modulus, and found no significant change. However, fiber-reinforced technopolymers and composites were not investigated in the study. Elraggal et al. also indicated that the presence of acid-containing media did not significantly impact the flexural strength of resin composite and hybrid ceramics [14]. Kim et al.’s study found no significant difference in mean flexural strength values and no significant difference in the results of statistical analyses for all materials after aging [42]. However, a study by Gülakar et al. (2023) showed a statistically significant difference in the mean flexural strength among ceramic CAD/CAM materials in response to 96 h of acid exposure [43]. Additionally, in a previous study, the effect of thermal cycling in water showed a significant decrease in both the flexural strength and modulus of a FRC CAD/CAM material intended for use in the fabrication of a metal-free framework for a restoration [12]. It would be of interest to further investigate the effect of acidic media on TDs following prolonged exposure, and compare this to their effect on ceramics.

The flexural modulus of CAD/CAM materials significantly impacts the durability of indirect restorations, especially in situations involving higher-than-normal occlusal loads and/or long-span FPDs [44,45]. Lower flexural moduli may enhance the flexibility and elasticity of dental restorations, but may also adversely affect their long-term stability [46]. Specifically, increased deformation under stress could lead to microfractures and marginal discrepancies, which will eventually compromise the integrity of final restorations [14,20,47]. Meanwhile, the flexural modulus results of the current study showed a significant change after immersion in the different media. This is in accordance with the results of a previous study by Scribante et al. [48], which tested the effect of immersion media on direct composites. Similarly, the effect of immersion of the composites in water on their flexural modulus was not statistically significantly different to that encountered with their immersion in acidic media. When FRCs are immersed in aqueous media, several mechanisms can lead to degradation of the material and a decrease in its physical and mechanical properties [6]. Hydrolysis reactions that weaken the bonds between the matrix and the reinforcing fibers may occur, reducing the overall structural integrity of the material [6,49,50]. The deterioration in the modulus following immersion in the different media, as seen in the current study, concurs with the results of a previous study that demonstrated the deterioration in hardness of an FRC CAD/CAM material in response to 7 days of immersion [18]. Similarly, thermocycling of an FRC (Everest C-Temp, KaVo Everest, Kaltenbach &Voigt GmbH, Biberach, Germany) caused softening due to a decrease in the flexural modulus, which may have been caused by water absorption in the resin matrix during thermocycling [12,51]. This reported deterioration in the modulus may eventually impact the success of restorative dental treatment.

The longitudinal design of the current in vitro study allowed for the observation of the immersion effect after a specified time under rigorously controlled standardized conditions, offering valuable insights the material’s behavior after intraoral use. This is especially beneficial in the investigation of newly developed dental materials [52]. It is important to acknowledge a few limitations in this study. The small sample size employed in the study might have limited the results. As is the case with in vitro studies, it is important to note that the created controlled environment cannot fully replicate the complexities of the oral environment, as variations in patient habits and intraoral conditions may affect outcomes differently. Only one CAD/CAM material was examined in this study, which may limit the generalizability of the results to other FRC materials. Therefore, future studies that include additional materials for comparison would be advantageous. Additionally, considering other mechanical properties and longer immersion periods with cyclic loading and fatigue testing could provide a more comprehensive understanding of the material. Further investigations into the effects of different finishing procedures, a longer immersion time, and exposure to other erosive acid media and beverages on the flexural properties of this technopolymer are recommended. The assessment of other mechanical and physical properties of the material in response to combined intraoral factors is recommended. Furthermore, incorporating microstructural analysis, like scanning electron microscopy, would enhance our understanding of how changes in mechanical properties relate to material degradation. This analysis could provide valuable insights into the underlying mechanisms of such changes, so further studies are recommended.

## 5. Conclusions

In conclusion, the immersion of a Trilor fiber-reinforced technopolymer for use in the CAD/CAM fabrication of definitive dental restorations was not significantly affected by exposure to various media for up to 48 h. This suggests that the flexural strength of indirect restorations made from the Trilor FRC CAD/CAM technopolymer can remain stable for up to 5 years, even with daily exposure to acidic media for a total of 90 min. However, the flexural modulus showed a significant decline in response to immersion, regardless of the solution’s pH, indicating some degree of softening of the tested materials. Further studies are necessary to validate these results and to compare them with those for other fiber-reinforced composite (FRC) dental CAD/CAM materials. Additionally, it is recommended to conduct wider-scale, longer-term studies to examine the materials’ responses to different immersion media, as well as to investigate surface and microstructural changes and explore other mechanical properties.

## Figures and Tables

**Table 1:** The material used in the study [11].

Material	Abb	Type/Chemical Composition	Manufacturer	Shade/Size	Flexural Modulus (GPa)	Flexural Strength (Mpa)
Trilor disk	TD	Pre-polymerized glass fiber-reinforced composite/ high-performance techno-polymer matrix with multi-directional glass fiber reinforcement; made up of about 74%wt of glass fibers	Bioloren, Saronno, Italy	FDS18 Ivory/18 mm disk	26 GPa	Biaxial 540 MPa

**Table 2:** The immersion media used in the study.

Material Name	pH	Contents	Manufacturer
Water	7	Deionized water	Advanced Technology Dental Research Laboratory (ADTRL), Faculty of Dentistry, King Abdulaziz University, Jeddah, Saudi Arabia
Artificial gastric acid	1.3	Deionized water (100 mL) and hydrochloric acid (20 mL)	Central Drug House (P) Ltd.—CDH, Delhi, India
Regular Coca-Cola	2.6	Carbonated water, sugar, caramel color, acidifier: phosphoric acid, natural cola flavors, caffeine.	Coca-Cola, Aujan Coca-Cola Beverages Company, Jeddah, Saudi Arabia

## Data Availability

The data presented in this study are available on request from the corresponding author.

## Abbreviations

The following abbreviations are used in this manuscript:

°C	Degrees Celsius
%wt	Weight percentage
ANOVA	Analysis of variance
ATDRL	The Advanced Technology Dental Research Laboratory
CAD/CAM	Computer-aided design/computer-aided manufacturing
$E_{flex}$	Flexural modulus
FEA	Finite element analysis
FPD	Fixed partial denture
FRC	Fiber-reinforced composite
$F_{s}$	Flexural strength
GPa	Gega Pascal
HCL	Hydrochloric acid
ISO	International Standards Organization
kN	Kilo Nuton
mm	Millimeter
mm/min	Millimeter/minute
MPa	Mega Pascal
SiC	Silicon carbide
TD	Trilor technopolymer disk (Bioloren, Saronno, Italy)
TDA	Trilor disk acid-immersion group
TDB	Trilor disk baseline group
TDC	Trilor disk cola-immersion group
TDW	Trilor disk water-immersion group
Tukey’s HSD	Tukey’s honestly significant difference
